# Case Report: Pathologic complete response to PRaG therapy in an elderly patient with refractory metastatic gastric cancer

**DOI:** 10.3389/fonc.2025.1726271

**Published:** 2026-01-15

**Authors:** Jun Lv, Fayi Yu, Linjiang Pan, Yeying Fang, Zhenghong Zhong, Xue Bai, Jinbiao Gao, Zhuxin Wei

**Affiliations:** Department of Radiotherapy, The First Affiliated Hospital of Guangxi Medical University, Nanning, Guangxi, China

**Keywords:** case report, metastatic gastric cancer, pathologic complete response (pCR), PRaG therapy, refractory

## Abstract

Hepatoid adenocarcinoma of the stomach (HAS) is a rare histologic subtype of gastric cancer with distinct pathological features and poor prognosis. Surgery and chemotherapy remain the primary treatment modalities, but outcomes are generally unsatisfactory. Radiotherapy can enhance tumor immunogenicity by releasing tumor antigens, thereby promoting antitumor immune responses and potentiating the efficacy of immune checkpoint inhibitors. We report a case of advanced HAS that progressed after first-line treatment with chemotherapy, immunotherapy, and targeted therapy. The patient subsequently received a combination of radiotherapy, programmed cell death protein 1 (PD-1) blockade, and granulocyte-macrophage colony-stimulating factor (GM-CSF)—termed PRaG therapy. The tumor demonstrated significant shrinkage following PRaG treatment and ultimately achieved pathologic complete response (pCR), with no serious adverse events aside from mild abdominal pain (NRS score 2). This case suggests that PRaG therapy may represent a promising therapeutic approach for patients with metastatic HAS.

## 1 Introduction

Hepatoid adenocarcinoma of the stomach (HAS) is a rare and aggressive subtype of gastric cancer, accounting for approximately 0.17% of all cases, with an annual incidence of 0.58–0.83 per million individuals (1). Characterized by frequent hepatic metastases, HAS portends a dismal prognosis, with reported 5-year survival rates as low as 8.3% to 9% (2, 3). No standardized treatment guidelines exist; management relies on extrapolation from case reports and limited evidence. Conventional surgery and chemotherapy offer minimal benefit, highlighting an urgent need for novel therapeutic strategies. Here, we describe a case of metastatic HAS in an elderly patient who achieved a remarkable and durable response following PRaG therapy, a novel combination of PD-1 blockade, Radiotherapy, and GM-CSF. This regimen leverages synergistic mechanisms: PD-1 inhibition reverses T-cell exhaustion (4), GM-CSF enhances dendritic cell maturation and antitumor immunity (5–7), and radiotherapy induces immunogenic cell death and potentially systemic abscopal effects (8).

## 2 Case description

### 2.1 Clinical presentation and initial diagnosis

**Figure 1:**
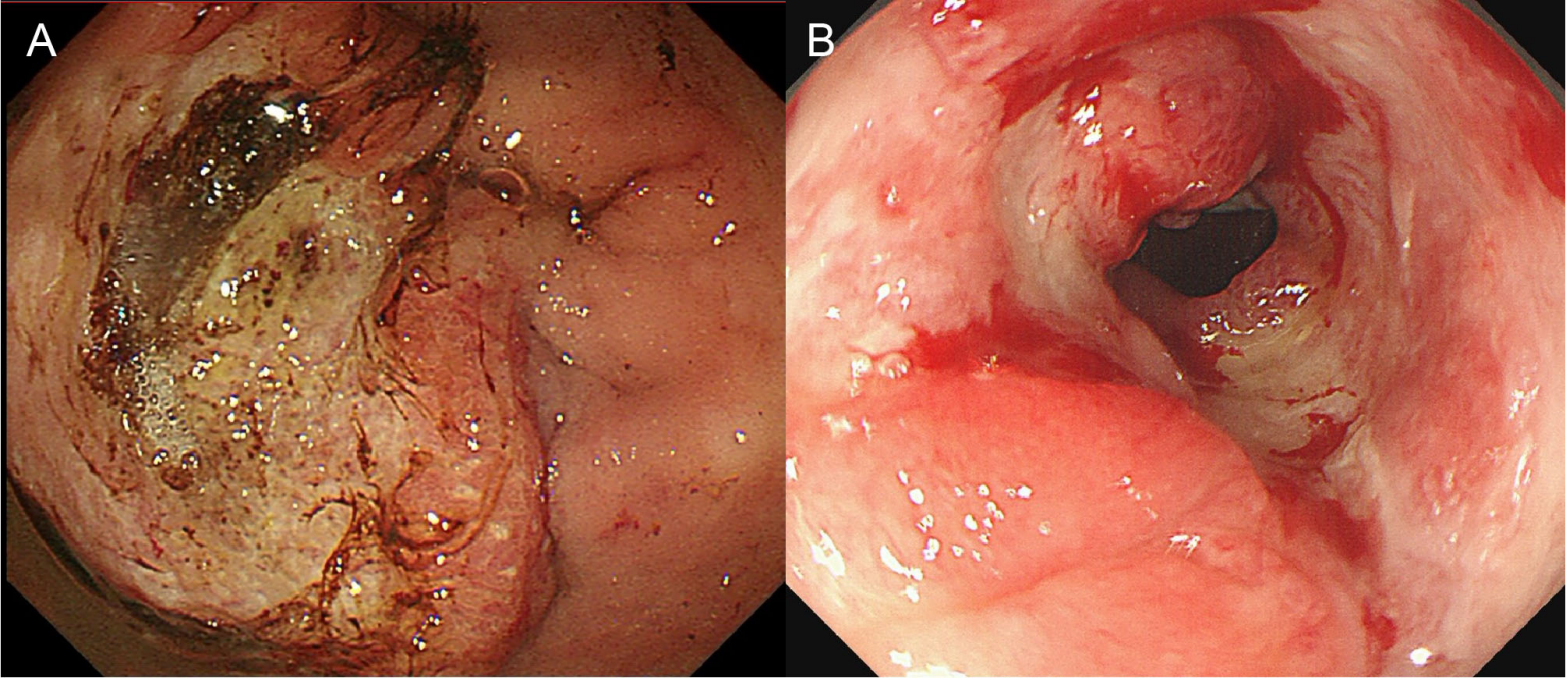
Endoscopic findings of the patient. **(A)** A large mass on the anterior wall of the gastric antrum with a superficial ulcer before treatment; **(B)** The gastric antral mass resolved following PRaG therapy.

**Figure 2:**
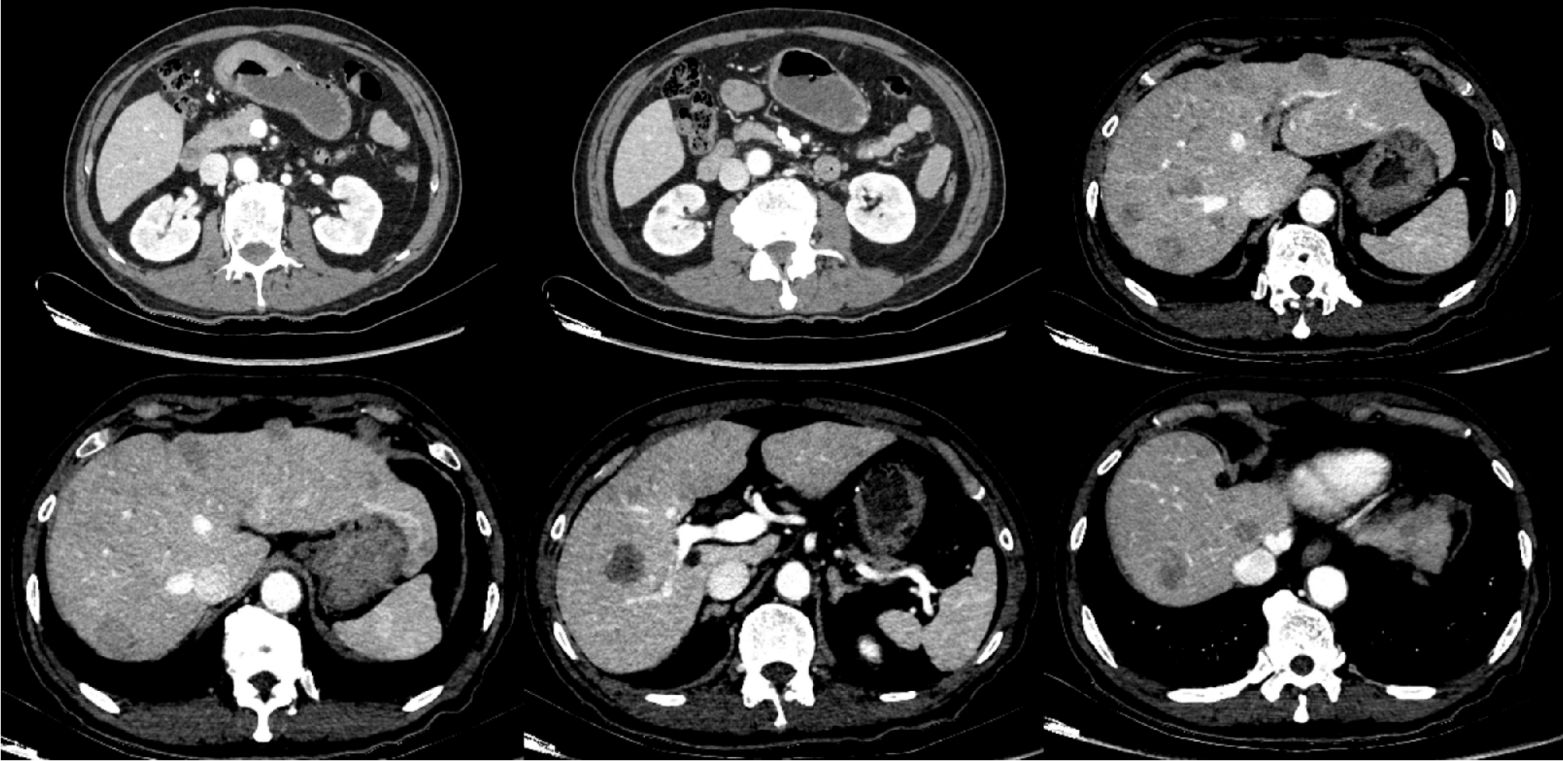
Pre-treatment CT scan demonstrating gastric antral wall thickening (findings suspicious for malignancy), retroperitoneal lymph node metastasis, and multiple liver metastases.

A 76-year-old man presented with melena and fatigue in November 2022. Upper endoscopy revealed a gastric antral mass (**Figure 1A**). Key diagnostic findings are summarized below. We have investigated the patient’s family and genetic information and confirmed that there is no family history of malignant tumors. The baseline diagnostic assessment and results after admission are as follows (**Table 1**; **Figure 2**).

**Table 1:** Baseline diagnostic workup and findings.

Investigation	Key results	Clinical implication
Histopathology & IHC	Hepatoid adenocarcinoma. IHC+: AFP, Glypican-3, Arginase-1. MSS, HER2 0, p53 mutant-type negative. Ki-67: 40%.	Confirmed HAS, aggressive phenotype.
Serum Tumor Marker	AFP: 47,040.09 ng/mL (normal: 0.89–8.78)	Indicator of tumor burden and monitoring.
Abdominal CT	Gastric antral wall thickening, multiple liver metastases, abdominal lymphadenopathy (**Figure 2**).	Confirmed Stage IVb disease (cT4bN1M1).

Given his poor performance status (ECOG 3), anemia (Hb 63 g/L), and advanced disease, he was deemed ineligible for surgery. The patient declined intensive chemotherapy.

### 2.2 Treatment timeline and therapeutic response

**Figure 3:**
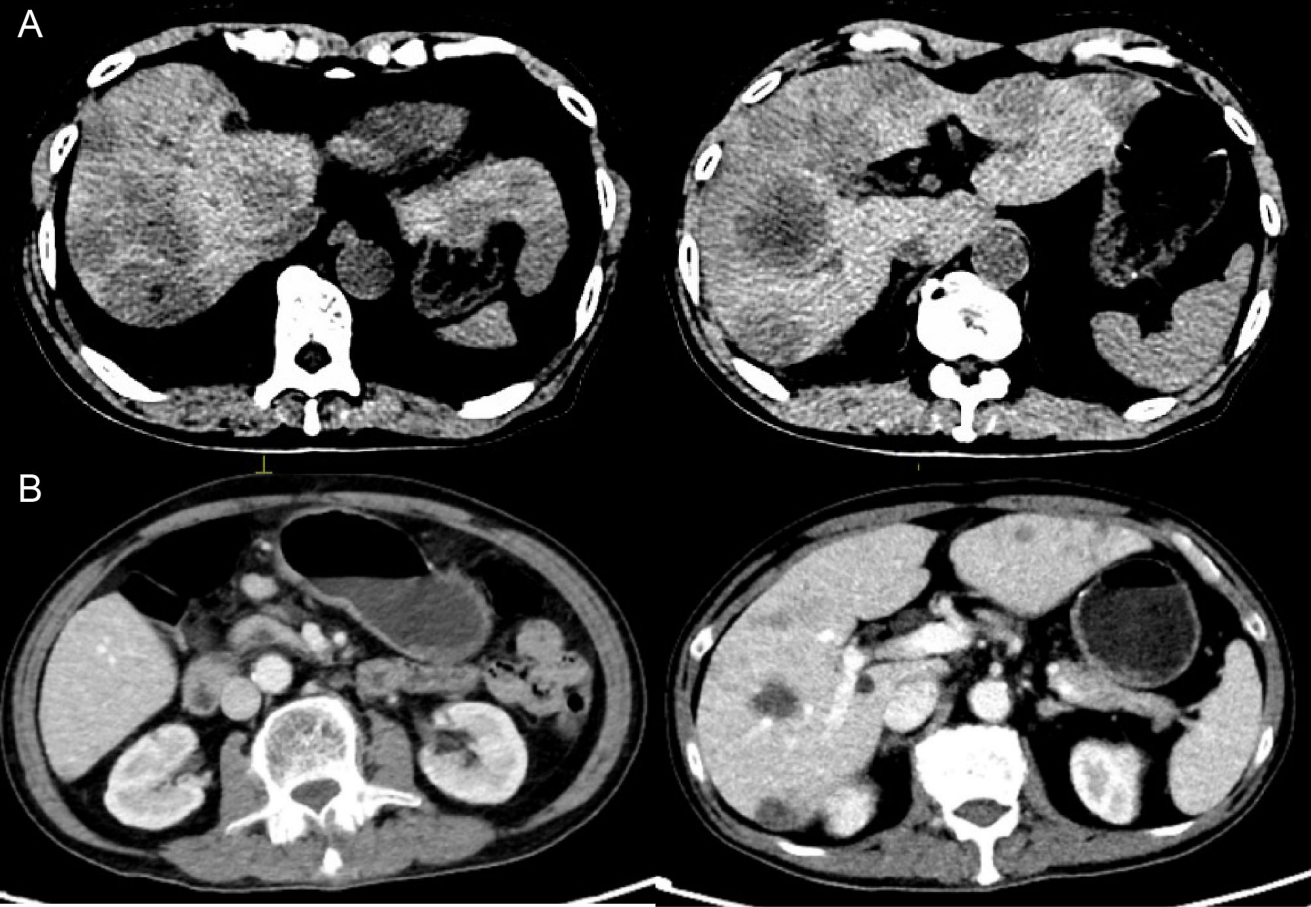
**(A)** Disease progression after triple-drug therapy with enlargement of liver metastases. **(B)** Partial response was achieved after two cycles of PRaG therapy, with a significant reduction in both primary and metastatic lesions.

**Figure 4:**
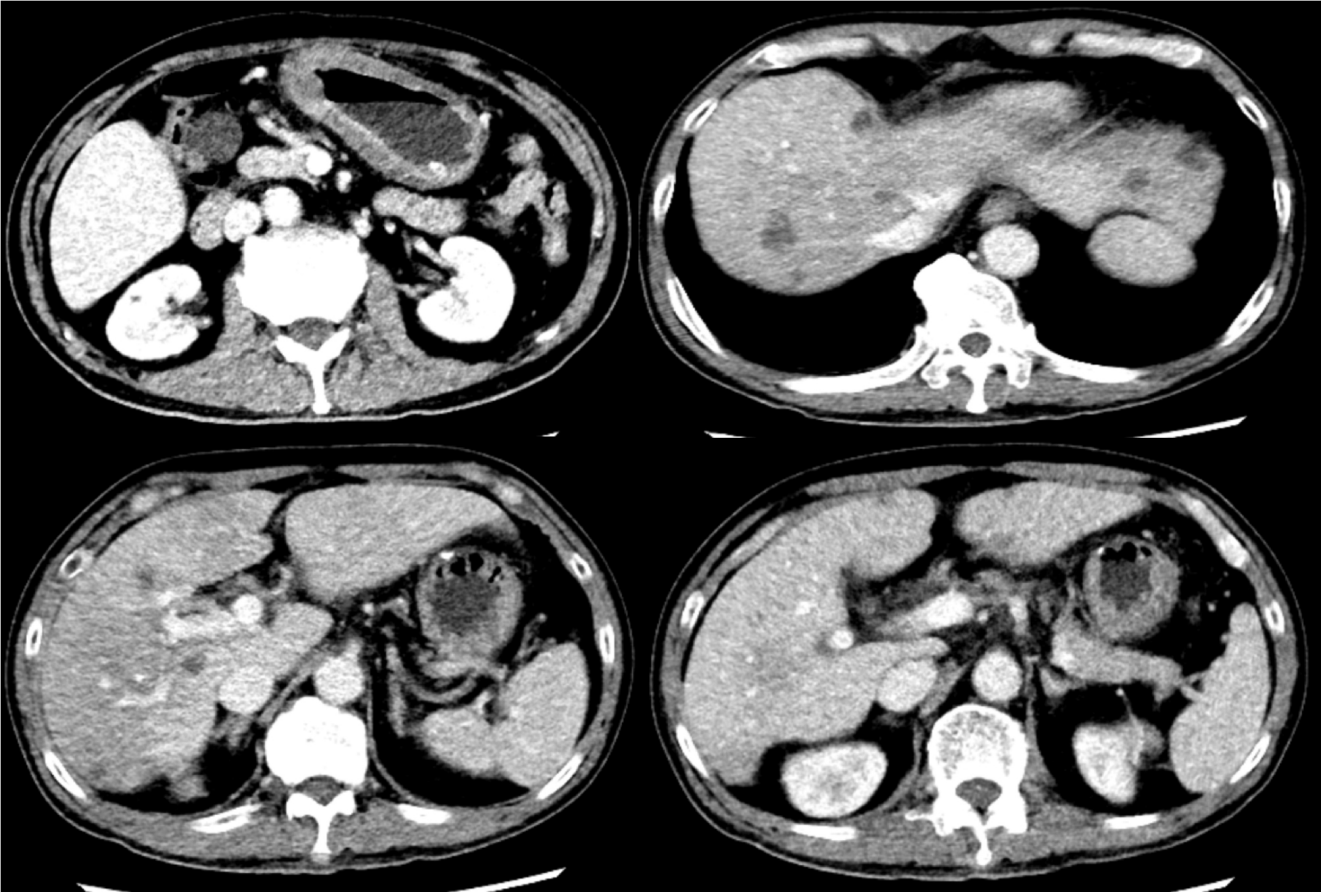
Partial response (PR) was achieved after 4 cycles of PRaG therapy, with continued tumor shrinkage on CT imaging.

Initial therapy with S-1, tislelizumab (PD-1 inhibitor), and apatinib failed, with rising AFP and radiographic progression after two cycles (**Figure 3A**). Subsequently, PRaG therapy was initiated. The treatment schedule and efficacy assessment at each stage are as follows (**Table 2**; **Figures 1B**, **3B**, **4**; **Supplementary Figure S1**).

**Table 2:** Treatment timeline and efficacy assessment.

Timepoint/phase	Intervention	Key imaging & lab findings	RECIST 1.1 response
Baseline (Nov 2022)	Diagnosis	Gastric mass, liver mets, AFP >47,000 ng/mL	–
Cycle 1-2 (Initial three-drug regimen)	S-1 + Apatinib + tislelizumab	Disease progression, AFP increased	Progressive Disease (PD)
PRaG Cycles 1-4	SBRT (Primary tumor and metastasis) + GM-CSF + tislelizumab + IL-2	After 2 cycles: Marked regression of all lesions. After 4 cycles: Continued regression, AFP decline.	Partial Response (PR) (**Figures 3B**, **4**)
Maintenance (Mar-Jul 2023)	tislelizumab monotherapy	Sustained response	Maintained PR
Treatment Holiday & Follow-up	All therapy discontinued	Nov 2024 (24 mos): Complete radiologic & pathologic resolution of primary and non-irradiated liver metastases (**Figure 1B**) (**Supplementary Figure S1**). AFP normalized.	Pathologic Complete Response (CR)

Treatment Details: Each 3-week PRaG cycle comprised SBRT (15 Gy/3 fx to gastric primary and lymph nodes in cycles 1-2; 24 Gy/3 fx to select liver metastases in cycles 3-4), subcutaneous GM-CSF (200 µg/day, days 1-7), tislelizumab (200 mg, day 5), and interleukin-2 (2 million IU/day, days 8-14).

Adverse Events: Post-PRaG, the patient experienced abdominal pain (NRS 6), attributed to radiation/immune-mediated gastritis, which led to discontinuation of combined therapy. Symptoms subsequently improved with analgesia.

Outcome: As of last follow-up (November 2024, 24 months from diagnosis), the patient maintained a pathologic complete response with excellent performance status and minimal analgesic requirement (NRS 2). Progression-free survival (PFS) was 24 months and ongoing.

## 3 Discussion

This case demonstrates an exceptional and durable pathologic complete response to PRaG therapy in a patient with treatment-naïve, AFP-positive metastatic HAS—a disease with a typically grave prognosis.

### 3.1 Contextualizing the outcome within existing literature

The efficacy observed here starkly contrasts with historical outcomes for metastatic HAS. Standard first-line chemotherapy for advanced gastric adenocarcinoma (e.g., fluoropyrimidine/platinum regimens) yields median overall survival (OS) of 8–14 months, with responses in HAS often being suboptimal and transient. The aggressive biology of HAS, frequently associated with AFP production and TP53 mutations as seen in our case, further contributes to chemoresistance (9–11). While immune checkpoint inhibitors (ICIs) have revolutionized treatment for some gastrointestinal cancers, their efficacy in microsatellite-stable (MSS) gastric cancer, including most HAS cases, remains limited as monotherapy. Our patient’s rapid progression on initial PD-1 inhibitor combination therapy underscores this limitation.

### 3.2 Mechanistic rationale for PRaG synergy and the abscopal effect

The profound response following PRaG therapy can be attributed to multimodal synergy. Radiotherapy, particularly SBRT, does not only locally ablate tumors but also induces immunogenic cell death, releasing tumor antigens and activating dendritic cells (DCs) (12, 16). The chosen hypofractionated schedules (e.g., 8 Gy x 3) are supported by preclinical data demonstrating optimal induction of type I interferon signaling and systemic immune activation compared to single high-dose or conventional fractionation (16–18). GM-CSF acts as a critical adjuvant in this setting by promoting the maturation and migration of antigen-presenting DCs, thereby bridging innate and adaptive immunity (19). Clinical studies in melanoma have shown that adding GM-CSF to immunotherapy can improve survival outcomes (20, 21). Concurrent PD-1 blockade prevents the inactivation of newly primed T-cells, enabling a sustained systemic attack. This synergistic triad likely enabled the observed abscopal effect—the complete regression of non-irradiated liver metastases—a rare phenomenon strongly suggestive of robust systemic antitumor immunity (13–15).

### 3.3 Clinical implications and future directions

This report provides proof-of-concept that combining radiotherapy with immune modulation (PD-1 blockade + GM-CSF) can overcome resistance in an aggressively chemorefractory tumor. It suggests that PRaG therapy warrants further investigation in selected patients with advanced HAS or other immunotherapy-resistant, MSS gastrointestinal malignancies. Future efforts must focus on identifying predictive biomarkers (beyond MSI/PD-L1) for such combinations, optimizing radiation dose/fractionation, and managing unique toxicity profiles like immune-mediated gastritis. Larger, prospective studies are needed to validate these findings and define the precise role of this multimodal approach in the therapeutic landscape.

## 4 Conclusion

We present a case of metastatic hepatoid adenocarcinoma of the stomach achieving a durable pathologic complete response with PRaG therapy after failing initial treatment. This outcome highlights the potential of combining radiotherapy with dual immune modulation (PD-1 inhibition and GM-CSF) to generate potent systemic antitumor immunity, even in traditionally immunotherapy-resistant tumors. While promising, this strategy requires validation in larger clinical trials to ascertain its efficacy, safety, and optimal application.

## Data Availability

The original contributions presented in the study are included in the article/**Supplementary Material**. Further inquiries can be directed to the corresponding author/s.

## Supplementary material

Supplementary Figure 1 Complete response (CR) was observed on CT imaging 16 months after cessation of anti-tumor therapy, with total resolution of the tumor.
