# Supplementary figures and images for: Case Report: Pathologic complete response to PRaG therapy in an elderly patient with refractory metastatic gastric cancer
Source: Front Oncol. 2026 Jan 15;15:1726271. doi: 10.3389/fonc.2025.1726271 (PMC12851978; doi:10.3389/fonc.2025.1726271)

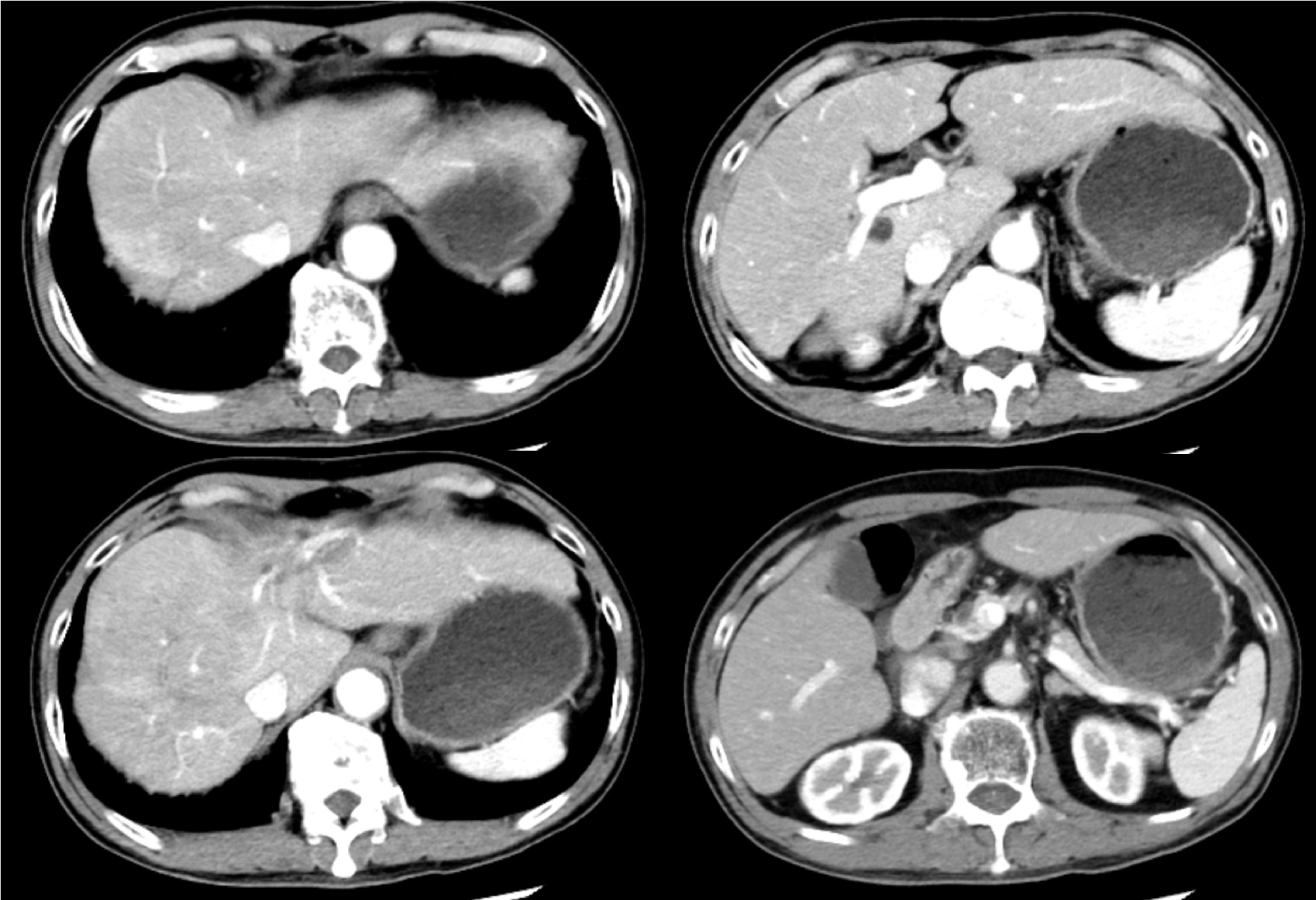

Supplement: Supplementary Figure 1 — Complete response (CR) was observed on CT imaging 16 months after cessation of anti-tumor therapy, with total resolution of the tumor. [file Image1.jpeg]
