# Supplementary material for: Case Report: Pathologic complete response to PRaG therapy in an elderly patient with refractory metastatic gastric cancer
Source: Front Oncol. 2026 Jan 15;15:1726271. doi: 10.3389/fonc.2025.1726271 (PMC12851978; doi:10.3389/fonc.2025.1726271)
Supplement: Supplementary file 2 [file Table1.docx]

**Diagnosis and Treatment Timeline**

| Timepoint | Diagnosis & Treatment Plan | Efficacy Evaluation |
| --- | --- | --- |
| 2022-11-13 | Pathologically diagnosed with gastric hepatoid adenocarcinoma | Stable Disease（SD） |
| 2022-11-25 to 2022-12-16 | 2 cycles of triple-drug combination therapy | Progressive Disease(PD) |
| 2022-12-26 to 2023-02-28 | cycles of PRaG regimen | Partial Response (PR) |
| 2023-03-28 to 2023-07-18 | 6 cycles of PD-1 inhibitor monotherapy as maintenance | Partial Response (PR) |
| 2024-11-21 to Present | Under follow-up | Complete Response（CR） |
